# Compact Modified Quatrefoil-Shaped Antenna with Dual-Circularly Polarized 28/38 GHz for 5G and Beyond Millimeter-Wave Applications

**DOI:** 10.3390/s26061890

**Published:** 2026-03-17

**Authors:** Asmaa E. Farahat, Khalid F. A. Hussein

**Affiliations:** Microwave Engineering Department, Electronics Research Institute, Cairo 11843, Egypt; asmaa@eri.sci.eg

**Keywords:** antenna, dual-circular polarized, millimeter-wave

## Abstract

This paper presents a compact dual-band circularly polarized (CP) antenna designed for millimeter-wave applications at 28 and 38 GHz, which are critical for emerging 5G and beyond wireless communication systems. The single-element antenna features an ultra-small radiating patch of size 3.34 mm × 3.34 mm and overall substrate footprint of 8 mm × 16 mm, implemented on a Rogers RO3003 substrate with a relative permittivity of 3 and thickness of 0.25 mm, making it highly suitable for space-constrained millimeter-wave front-end integration. Circular polarization is successfully achieved at both bands, with measured axial ratios of 1.4 dB at 28 GHz and 2.2 dB at 38 GHz. Surface current distribution is thoroughly analyzed at both frequencies, showing proper rotation and confirming the antenna’s ability to generate strong circular polarization. The antenna also exhibits high radiation efficiency (~87% at 28 GHz and ~82% at 38 GHz) and peak realized gains of 7.5 dBi and 5.5 dBi, respectively. Measured results demonstrate excellent impedance matching, stable radiation patterns, and strong agreement with simulations. The combination of compact size, robust CP performance, and efficient radiation makes the proposed antenna a promising candidate for circularly polarized millimeter-wave systems, including 5G base stations, user equipment, and future high-frequency wireless platforms.

## 1. Introduction

The 28 GHz and 38 GHz millimeter-wave bands are widely regarded as key spectrum resources for 5G and next-generation wireless systems because they provide large contiguous bandwidths capable of supporting extremely high data rates, low transmission latency, and increased network capacity. These bands have been internationally standardized and allocated in many regions for early 5G deployment, making them suitable for both outdoor cellular base stations and indoor high-speed wireless services. Despite these advantages, communication at mm-wave frequencies faces several propagation constraints, including substantial free-space path loss, attenuation due to atmospheric absorption, and strong sensitivity to blockage and shadowing. Consequently, antenna designs targeting the 28/38 GHz bands must achieve a balance between compact physical dimensions, adequate gain, stable radiation performance, and high efficiency, while also remaining suitable for integration within the space-limited architectures of modern wireless devices [1,2,3].

Circularly polarized (CP) antennas have attracted considerable interest for millimeter-wave wireless applications. Compared with linearly polarized antennas, CP designs provide several practical advantages, such as minimizing polarization mismatch, improving resistance to multipath effects, and reducing the sensitivity to the relative orientation between transmitting and receiving antennas. These characteristics are especially important for devices operating at 28 GHz and 38 GHz, including mobile terminals, user equipment, and small-cell base stations, where variations in device positioning and reflections from surrounding objects may noticeably affect link reliability. Therefore, CP antennas are regarded as a suitable solution for achieving stable mm-wave communication performance in practical 5G and future wireless networks [4,5]. Moreover, circular polarization is beneficial for advanced multi-antenna architectures, including massive MIMO and beamforming systems, where the use of polarization diversity can improve channel reliability and increase overall communication capacity.

To date, very few studies have demonstrated simultaneous dual-band circular polarization at both 28 and 38 GHz. Most reported mmWave antennas focus on single-band CP or dual-band linear polarization.

Recent research efforts have focused on realizing compact dual-band CP antennas covering the 28 GHz and 38 GHz bands while maintaining low axial ratio, sufficient gain, and ease of integration. Various techniques have been reported, including modified microstrip patch geometries, dielectric resonator antennas (DRAs), defected ground structures, and metasurface-assisted designs to generate circular polarization at one or both mmWave bands [6,7,8]. In addition, several works have demonstrated dual-band or multi-band CP antenna arrays operating at 28/38 GHz for beamforming and high-capacity 5G applications, emphasizing the importance of polarization purity and compact size. Despite these advances, achieving dual-band circular polarization within an ultra-compact footprint remains a challenging task, motivating further research on miniaturized CP antenna structures suitable for future mm-Wave wireless devices.

Several antenna designs have been reported in the literature to address dual-band operation at 28 and 38 GHz for millimeter-wave communication systems, each adopting different techniques to balance compactness, polarization, gain, and efficiency. In ref. [9], the authors proposed a compact dual-band antenna operating at 28 GHz and 38 GHz with circular polarization achieved through geometrical perturbations in the radiating structure. The design focused on maintaining a small footprint while enabling dual-band CP characteristics using a single-feed configuration. The antenna demonstrated acceptable impedance matching and axial-ratio performance at both bands, with moderate realized gain and radiation efficiency, particularly at the higher frequency band. A dual-band circularly polarized antenna specifically targeting millimeter-wave 5G applications is presented in ref. [10]. Circular polarization was realized at both 28 GHz and 38 GHz by introducing asymmetry in the patch geometry, which generated two orthogonal modes with a 90° phase difference. The work mainly emphasized polarization characteristics and axial-ratio bandwidth, while quantitative radiation performance metrics such as gain and efficiency were not explicitly reported. Nevertheless, the design successfully demonstrated stable CP behaviour at both operating bands. In ref. [11], an Artificial Magnetic Conductor (AMC)/metasurface-backed antenna is introduced to enhance gain and axial-ratio bandwidth for dual-band operation covering the 28 GHz and 38 GHz ranges. The use of an artificial magnetic conductor significantly improved forward radiation and gain, especially at the higher band. However, this enhancement came at the cost of increased antenna size, higher profile, and additional fabrication complexity. The best results showed high realized gain at both bands, particularly when the AMC layer was employed. A compact dual-band microstrip patch antenna employing defected ground structures to achieve operation at 28 GHz and 38 GHz is proposed in ref. [12]. The antenna was linearly polarized and optimized for high radiation efficiency and gain. Very high efficiency and relatively high realized gain were reported at both bands; however, the absence of circular polarization makes the design more sensitive to polarization mismatch in practical mobile and user-equipment scenarios. In ref. [13], a dual-band antenna designed primarily for MIMO millimeter-wave systems was reported. The antenna operated at 28 GHz and 38 GHz with linear polarization and demonstrated high radiation efficiency and strong realized gain, particularly at the upper band. While the design offered robust performance and suitability for MIMO configurations, it required a larger footprint compared to more compact single-element CP antennas.

Overall, these studies demonstrate that while high gain and efficiency can be achieved using larger apertures, metasurfaces, or linear polarization, achieving dual-band circular polarization with compact size and clearly reported radiation performance remains challenging, motivating further investigation in this area.

This paper presents a compact dual-band circularly polarized (CP) antenna operating at 28 and 38 GHz, two key millimeter-wave bands for emerging 5G and beyond wireless systems, including mobile access and fixed wireless backhaul. By integrating both bands within a single ultra-compact footprint of only 8 mm × 16 mm on a Rogers RO3003 substrate (εr = 3, thickness = 0.25 mm), the proposed design enables high spectrum flexibility and seamless integration into space-constrained mmWave front-end modules. Unlike conventional single-band or linearly polarized counterparts, the antenna achieves robust circular polarization at both frequencies, with measured axial ratios of 1.4 dB at 28 GHz and 2.2 dB at 38 GHz, thereby mitigating polarization mismatch and enhancing link reliability under device rotation and misalignment. The generation of strong CP is rigorously validated through surface current distribution analysis, confirming proper orthogonal mode excitation and rotation at both bands. Despite its significantly reduced size, the antenna maintains high radiation efficiency (~87% at 28 GHz and ~82% at 38 GHz) and competitive peak realized gains of 7.5 dBi and 5.5 dBi, demonstrating a favorable trade-off between miniaturization and radiation performance. Measured results show excellent impedance matching, stable radiation characteristics, and strong agreement with simulations. The combination of dual-band operation, high-efficiency circular polarization, and ultra-compact dimensions highlights the novelty and practical advantage of the proposed antenna for compact beamforming arrays, 5G user equipment, base stations, and future high-frequency wireless platforms.

## 2. Circularly Polarized Modified Quatrefoil Antenna Design

A thin, low-loss substrate is chosen to suit the planar configuration of the proposed antenna and its operation in the millimeter-wave band. The antenna is fabricated on a Rogers RO3003™ dielectric material with a thickness of 0.25 mm, relative permittivity of 3, and a loss tangent of 0.001. The antenna geometry and the underlying design methodology are described in detail. Due to the sensitivity of antenna performance to its physical dimensions, the design procedure involves comprehensive parametric optimization using the full-wave electromagnetic simulation software CST Microwave Studio 2024 to determine the optimal dimensional values. This process ensures efficient operation across the two target frequency bands while maintaining an axial ratio below 3 dB, which is required to achieve high-quality circular polarization in both bands.

To realize circular polarization, the proposed antenna employs a quatrefoil-shaped radiator, whose inherent geometric symmetry supports the generation of circularly polarized fields. In the proposed modified quatrefoil radiator, two orthogonal degenerate modes can be supported within the radiating structure. Circular polarization is obtained by properly perturbing the quatrefoil geometry, such that these two modes are excited with equal amplitudes. The introduced modifications break the structural symmetry in a controlled manner, leading to the excitation of two modes with mutually orthogonal electric-field orientations. One mode exhibits even symmetry, while the other shows odd symmetry with respect to the principal diagonal of the modified quatrefoil. By carefully tuning the geometric parameters of the modified quatrefoil, the required 90° phase difference between the two modes is achieved, resulting in the generation of circular polarization. The design stages are explained in detail in the following subsections.

Four parasitic elements are placed around the modified quatrefoil-shaped patch and capacitively coupled to it through narrow gaps. This coupling is used to effectively tune the resonant frequencies of the two radiating modes supported by the modified quatrefoil radiator. In addition, narrow slits are introduced in the ground plane to further control the current distribution and enhance impedance matching and polarization performance.

**Figure 1:**
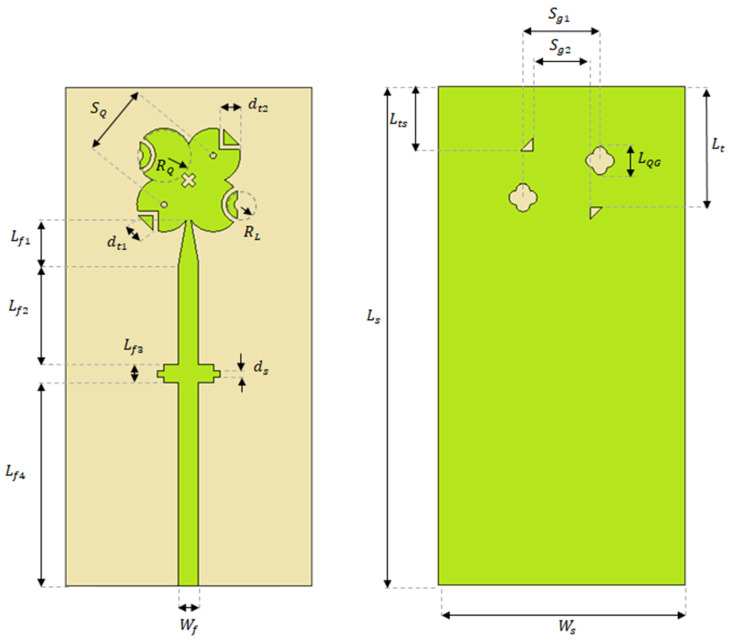
Geometry of the proposed modified quatrefoil-shaped antenna structure.

Impedance matching at both resonant frequencies is realized through careful optimization of the modified quatrefoil patch dimensions, the ground-plane cuts, the four parasitic elements, and the coupling-gap width between the parasitic elements and the main radiator. An inset-fed configuration is intentionally avoided, as it would break the diagonal symmetry of the antenna and negatively impact the circular polarization performance. Instead, the antenna is excited using a tapered microstrip feed line, which preserves structural symmetry while providing effective excitation and input-impedance matching. The tapered microstrip line has a width of 0.64 mm at the feed point. The complete geometry of the proposed antenna, including all relevant structural features, is illustrated in Figure 1, while the optimized values of the corresponding dimensional parameters used in the design are summarized in Table 1.

### 2.1. Design Stages

**Figure 2:**
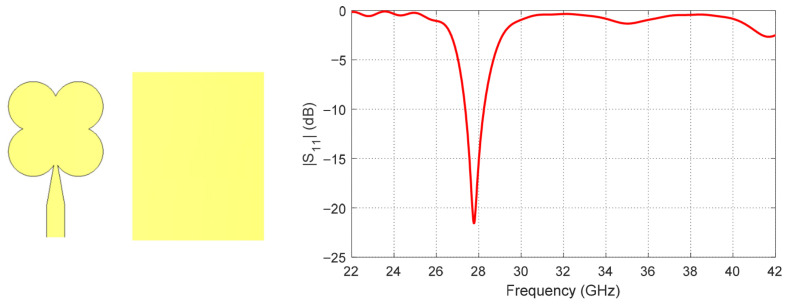
Reflection coefficient (*S*_11_) of the initial quatrefoil-shaped patch (stage 1), showing good matching at 28 GHz and no matching at 38 GHz.

The proposed antenna is developed through three main design stages to achieve dual-band operation and circular polarization at 28 GHz and 38 GHz. In the first stage, a resonant radiating patch is designed to operate at 28 GHz using a quatrefoil-shaped geometry. This shape is selected to provide the required fundamental resonance while maintaining a compact footprint suitable for millimeter-wave applications. The initial design focuses on establishing the primary resonance at 28 GHz with acceptable impedance characteristics. The reflection coefficient (*S*_11_) corresponding to this first design stage is presented in Figure 2.

**Figure 3:**
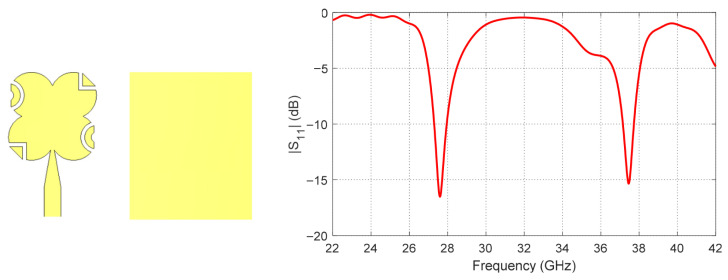
Reflection coefficient (*S*_11_) after adding diagonally symmetric parasitic elements (stage 2), showing improved matching near both 28 GHz and 38 GHz.

In the second stage, parasitic elements are introduced to enable operation at the higher-order resonant mode around 38 GHz. These parasitic elements are added with diagonal symmetry around the main patch by cutting portions of the radiating patch and inserting capacitive loading sections. This arrangement not only improves the impedance matching of the higher-order mode but also begins to enhance the diagonal symmetry of the structure, which is beneficial for generating circular polarization at both operating bands. The resulting *S*_11_ performance after introducing these diagonally symmetric parasitic elements is shown in Figure 3.

**Figure 4:**
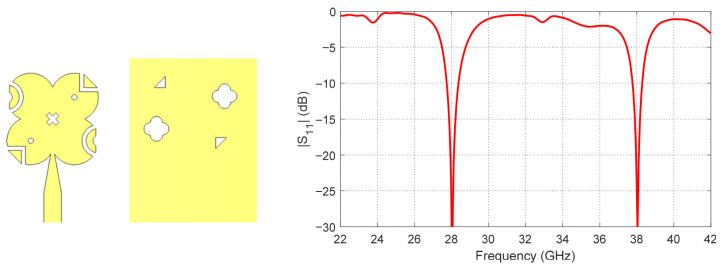
Reflection coefficient (*S*_11_) of the final design (stage 3) with ground perforations and top slots, demonstrating nearly perfect matching at both operating frequencies.

**Figure 5:**
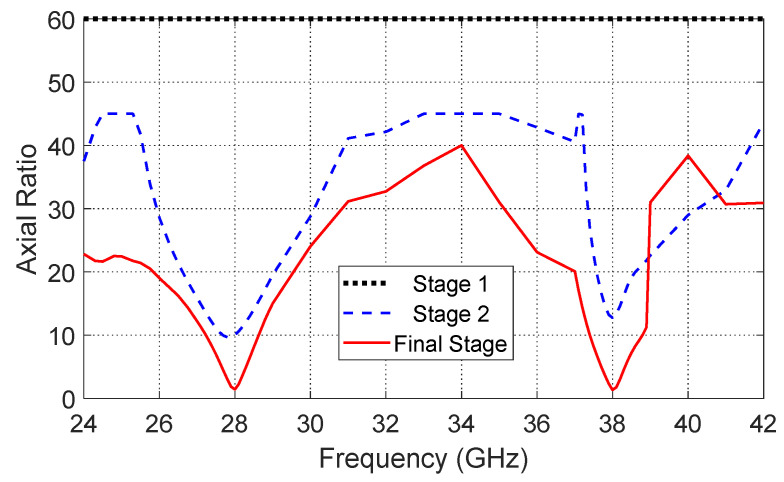
Axial ratio comparison for all three design stages, illustrating the evolution from linear polarization (AR > 60) in stage 1 to improved circular polarization (~AR 10) in stage 2, and excellent circular polarization (AR < 2) in stage 3.

In the third stage, further structural modifications are implemented to strengthen circular polarization performance. Specifically, ground perforations are introduced in the ground plane, and additional slots are etched on the top radiating surface. These modifications further increase the diagonal symmetry of the antenna structure, facilitating the excitation of two orthogonal modes with the required phase difference, resulting in good circular polarization at both 28 GHz and 38 GHz. The reflection coefficient for the final design stage is illustrated in Figure 4. Additionally, Figure 5 presents the axial ratio for the three design stages in a single plot, showing the progressive improvement in circular polarization achieved through these successive design enhancements.

By observing Figure 2, Figure 3, Figure 4 and Figure 5, it is clear that Figure 2 shows the reflection coefficient (*S*_11_) of the initial patch design, indicating good matching at 28 GHz, while no significant matching is observed at 38 GHz. After introducing the diagonally symmetric parasitic elements in stage two, Figure 3 demonstrates improved impedance matching near both 28 GHz and 38 GHz. In the final stage, with the addition of ground perforations and slots on the top patch, Figure 4 shows nearly perfect matching at both operating frequencies. By comparing the axial ratio in Figure 5, it is evident that the initial antenna exhibits linear polarization with very high AR values exceeding 60. The addition of the diagonally symmetric parasitic elements improves the AR around the two operating bands, reducing it to approximately 10, though it is still above the acceptable circular polarization threshold (3 dB). With the final modifications, including the slots in the ground plane and on the patch, the axial ratio drops below 2 at both bands, indicating excellent circular polarization performance. Additionally, the impedance matching at both frequencies is further enhanced by the use of a tapered transmission line feeding technique, which optimizes the coupling between the feed and the radiating structure.

### 2.2. Parameter Analysis and Impact on Antenna Performance

A comprehensive parameter analysis was conducted to understand the impact of each structural and feeding element on the antenna’s dual-band performance and circular polarization characteristics. The geometry of the quatrefoil patch primarily determines the fundamental resonance at 28 GHz, with its dimensions directly affecting the resonance frequency and input impedance of the lower band. The parasitic elements, including their size, shape, and precise location around the main patch, play a critical role in exciting the higher-order resonance near 38 GHz and in tuning the impedance matching of this second band. The diagonal symmetry of both the parasitic elements and the slots introduced on the top patch and ground plane is a key factor in achieving high-quality circular polarization at both operating frequencies, as it governs the generation of orthogonal modes with the required phase difference, thus controlling the axial ratio. Additionally, the transmission line feeding parameters, including its length, tapering profile, and position relative to the patch, are carefully optimized to enhance impedance matching across both bands, ensuring efficient power transfer from the feed to the radiating structure. This parameter study highlights the interdependent roles of the patch geometry, parasitic elements, diagonal symmetry, and feeding design in achieving the targeted dual-band circularly polarized operation.

## 3. Modified Quatrefoil Fabrication and Measured Performance

### 3.1. Fabrication Process

**Figure 6:**
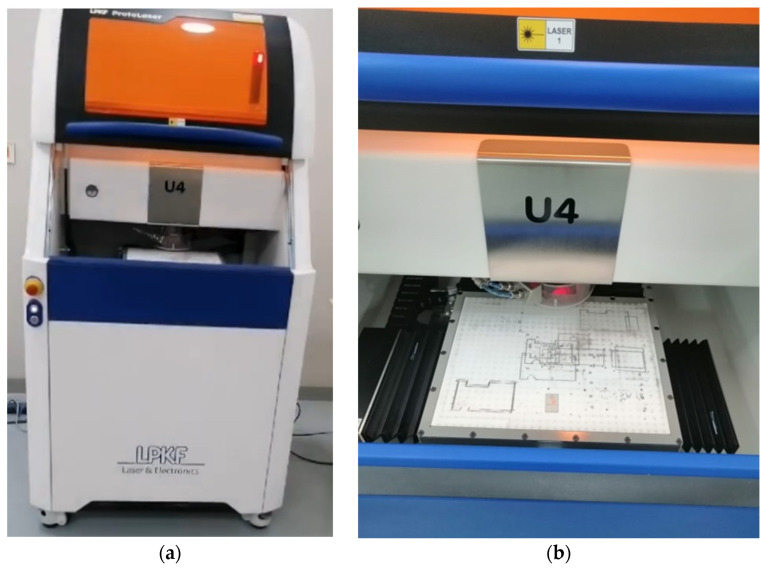
The LPKF ProtoLaser U4 machine used in this work to fabricate the proposed antenna. (**a**) The LPK machine. (**b**) Zoomed view of the working area.

The LPKF ProtoLaser U4 system (LPKF company, Garbsen, Germany), shown in Figure 6, was employed to fabricate the proposed antenna. It is a laser-based PCB prototyping platform designed for high-precision microwave and millimeter-wave circuit fabrication. The system uses a finely focused ultraviolet laser beam to selectively ablate copper, enabling direct patterning of conductive traces without requiring chemical etching. This non-contact laser structuring ensures minimal thermal stress on the substrate, preserving its dielectric properties.

The ProtoLaser U4 provides a minimum track width of approximately 50 µm and minimum spacing of 13 µm, achievable with a ~20 µm laser spot size. These capabilities are essential for fabricating the fine transmission lines, narrow gaps, and accurate slot geometries for the antenna design operating in the mm-wave band. A key advantage of the system is its suitability for rapid prototyping and iterative optimization. By eliminating wet chemical processes, the LPKF machine ensures a clean, fast, and environmentally friendly fabrication process. Furthermore, its ability to achieve sub-50 µm precision enables reliable integration of multilayer structures, such as the antenna, defected ground plane, and biasing circuits, while minimizing misalignment and parasitic effects. These features make the LPKF ProtoLaser U4 particularly well-suited for the development of advanced high-frequency antenna prototypes.

**Figure 7:**
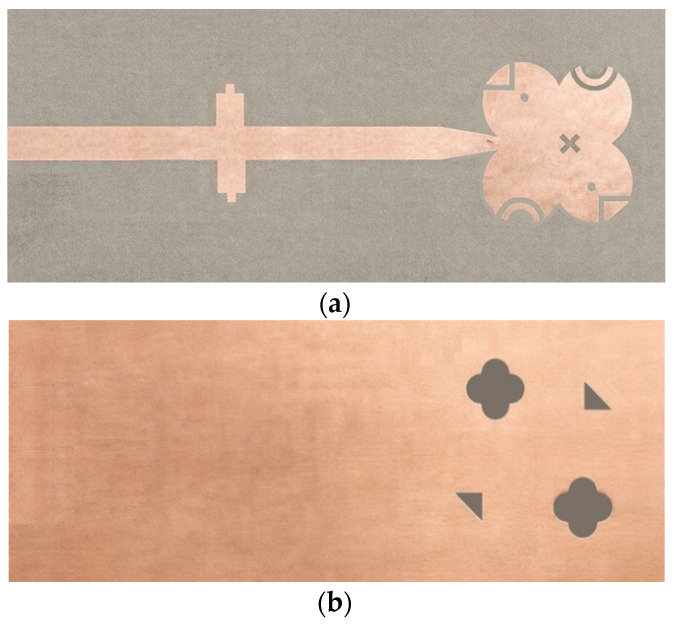
(**a**) Front and (**b**) back views of the fabricated modified quatrefoil-shaped antenna.

Figure 7 presents the fabricated antenna with both front and back views, highlighting the realized patch, parasitic elements, and ground-plane features.

### 3.2. Measurement of Reflection Coefficient

**Figure 8:**
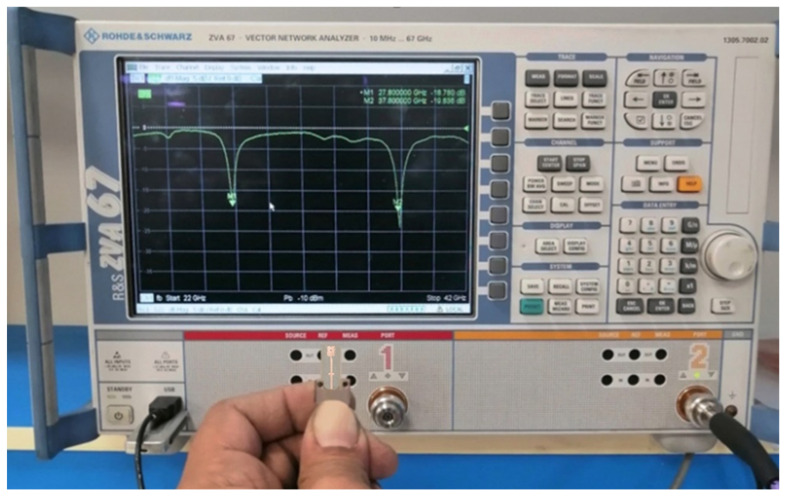
Measurement setup showing the connection of the fabricated antenna to the ZVA67 vector network analyzer.

**Figure 9:**
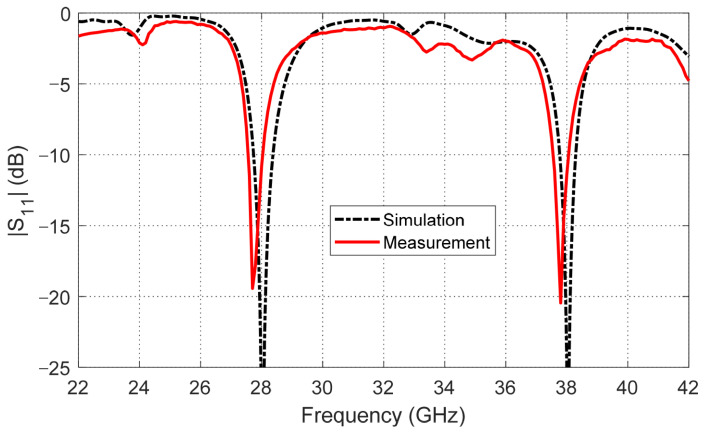
Simulated and measured reflection coefficient (*S*_11_) of the antenna, showing resonances at 28 GHz and 38 GHz.

The proposed modified quatrefoil-shaped antenna has been successfully fabricated on the Rogers RO3003™ substrate, and its reflection coefficient ($S_{11}$) was measured to validate the simulated design. Measurements were performed using a Rohde & Schwarz ZVA67 vector network analyzer (VNA), ensuring precise characterization over the intended frequency range. The measurement setup, showing the connection of the antenna to the VNA via the tapered microstrip feed, is illustrated in Figure 8. The measured $S_{11}$ response, depicted in Figure 9, exhibits two clear resonant minima at approximately 28 GHz and 38 GHz, corresponding to the target frequency bands. A direct comparison with the simulated results shows very good agreement, confirming the effectiveness of the design in achieving dual-band operation with proper impedance matching.

### 3.3. Measurement of the Gain and Radiation Patterns

The radiation pattern and gain of an antenna can be characterized through experimental measurements using two complementary setups. In one approach, a vector network analyzer (VNA) can be employed. In a second approach, a vector signal generator (VSG) and vector signal analyzer (VSA) can be used to transmit and receive a known signal, enabling the direct measurement of the radiated field and far-field radiation pattern. In this study the VSG and VSA are selected to measure the radiation characteristics of the proposed antenna.

The equipment, tools, and components used for experimental setup of the gain and radiation patterns are listed in Table 2.

**Figure 10:**
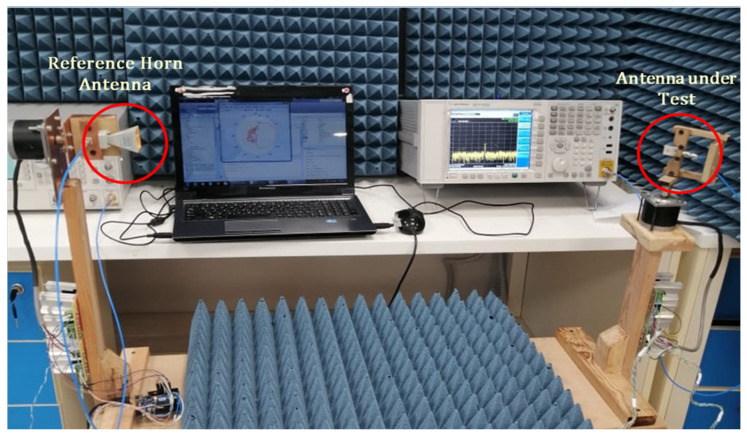
Measurement of the far-field patterns of the proposed antenna using the VSG and VSA.

For gain and normalized pattern measurement, the reference horn antenna boresight is directed towards the antenna under test (AUT) as shown in Figure 10. The AUT is mounted on a rotator that can be used to rotate the antenna about its axis in its horizontal and elevation planes. Let the distance between the reference horn antenna and the AUT be D. It should be noted that, during gain and pattern measurement, the AUT should be in the far field region of the reference horn antenna. The far-field distance can be calculated according to the formula $2L^{2}/\lambda$, where L is largest dimension of the antenna and λ is the shortest wavelength during measurement. As the largest dimension of the reference horn antenna is 5 cm and the highest frequency is 40 GHz, the far-field distance, according to the above formula, is 100 cm. For measurement, the AUT is connected to port 1 of the VNA and the reference horn antenna is connected to port 2 through flexible coaxial cables of low insertion loss, or alternatively, the AUT can be connected to VSG and the reference horn antenna connected to VSG.

Let the positive z-axis be in the outward direction normal to the plane of the printed antenna and $\left(\theta ,\phi \right)$ be angular dimensions of the corresponding spherical coordinate system. The power density at the reference receiving antenna can be expressed as follows:

(1) $$\frac{{\left|E_{R}\left(\theta ,\phi \right)\right|}^{2}}{2\eta }=\frac{P_{T}}{4\pi D^{2}}G_{T}\left(\theta ,\phi \right)$$

where $P_{T}$ is the transmitted power, $E_{R}\left(\theta ,\phi \right)$ is the electric field at the receiving antenna when the transmitting antenna is oriented in the direction $\left(\theta ,\phi \right)$, $G_{T}\left(\theta ,\phi \right)$ is the gain of the transmitting antenna (AUT) in the same direction, and η=120π Ω is the intrinsic impedance of free space.

The power received by the reference horn antenna when the transmitting antenna is oriented in the direction $\left(\theta ,\phi \right)$ can be expressed as follows:

(2) $$P_{R}\left(\theta ,\phi \right)=\frac{{\left|E_{R}\left(\theta ,\phi \right)\right|}^{2}}{2\eta }A_{R}=\frac{P_{T}}{4\pi D^{2}}G_{T}\left(\theta ,\phi \right)\frac{\lambda^{2}}{4\pi }G_{R}$$

where $A_{R}=\frac{\lambda^{2}}{4\pi }G_{R}$ and $G_{R}$ are, respectively, the effective area and the gain of the reference horn antenna in the boresight direction.

Using (2), the gain of the AUT can be expressed as follows:

(3) $$G_{T}\left(\theta ,\phi \right)={\left(\frac{4\pi D}{\lambda }\right)}^{2}\frac{P_{R}\left(\theta ,\phi \right)}{P_{T}}G_{R}$$

If the gain pattern is measured using the measurement setup that employs the VSG and VSA as shown in Figure 10, the $P_{R}\left(\theta ,\phi \right)$ can be obtained from the readings of the VSA when the AUT is oriented in the direction $\left(\theta ,\phi \right)$ and the $P_{T}$ is the transmitted power that is set by the control of the VSG.

If the VNA is used for measurement, the ratio $P_{R}\left(\theta ,\phi \right)/P_{T}$ can be obtained from the S-parameter $S_{21}\left(\theta ,\phi \right)$ when the transmitting antenna is oriented in the direction $\left(\theta ,\phi \right)$ as follows:

(4) $$\frac{P_{R}\left(\theta ,\phi \right)}{P_{T}}={\left|S_{21}\left(\theta ,\phi \right)\right|}^{2}$$

Thus, the gain pattern $G_{T}\left(\theta ,\phi \right)$ of the AUT can be calculated from the readings $S_{21}\left(\theta ,\phi \right)$ (obtained by the VNA) as follows:

(5) $$G_{T}\left(\theta ,\phi \right)={\left(\frac{4\pi D}{\lambda }\right)}^{2}G_{R}{\left|S_{21}\left(\theta ,\phi \right)\right|}^{2}$$

The normalized radiation pattern of the AUT can be obtained as follows. The electric field at the receiving antenna when the AUT is oriented in the direction $\left(\theta ,\phi \right)$ can be obtained as follows:

(6) $$\left|E_{R}\left(\theta ,\phi \right)\right|=\sqrt{2\eta P_{R}\left(\theta ,\phi \right)}$$

Substituting from (4) into (6), the following expression is obtained:

(7) $$\left|E_{R}\left(\theta ,\phi \right)\right|=\sqrt{2\eta P_{T}}\left|S_{21}\left(\theta ,\phi \right)\right|$$

Thus, the normalized radiation pattern can be obtained from the readings of the VNA as follows:

(8) $$\left|{\hat{E}}_{R}\left(\theta ,\phi \right)\right|=\frac{\left|E_{R}\left(\theta ,\phi \right)\right|}{{\left|E_{R}\right|}_{max}}=\frac{\left|S_{21}\left(\theta ,\phi \right)\right|}{{\left|S_{21}\right|}_{max}}$$

where ${\left|E_{R}\right|}_{max}$ is the maximum of $\left|E_{R}\left(\theta ,\phi \right)\right|$ over all the spatial directions $\left(\theta ,\phi \right)$ at the operating frequency and ${\left|S_{21}\right|}_{max}$ is the maximum of $\left|S_{21}\left(\theta ,\phi \right)\right|$ at the same frequency.

**Figure 11:**
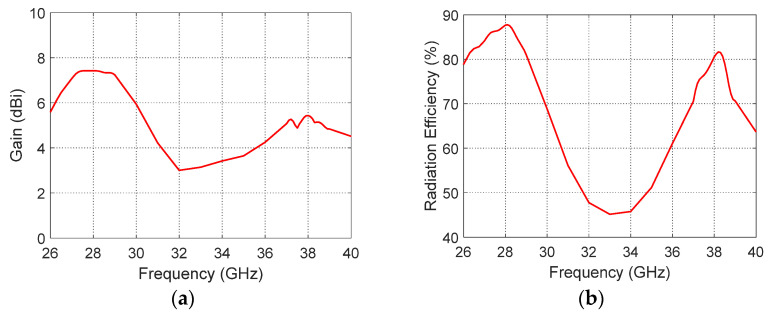
(**a**) Maximum gain of the proposed modified quatrefoil-shaped antenna across the operating frequency range. (**b**) Radiation Efficiency.

The radiation performance of the proposed modified quatrefoil-shaped antenna was comprehensively evaluated in terms of both its maximum gain and far-field radiation characteristics to ensure that it satisfies the requirements of dual-band millimeter-wave applications. The maximum gain curve of the antenna, shown in Figure 11a, demonstrates the gain variation over the operating frequency range, with distinct peaks observed at the two resonant frequencies. Specifically, the antenna achieves a peak gain of 7.5 dB at 28 GHz and 5.5 dB at 38 GHz. These peaks indicate efficient radiation and effective power transfer at both frequency bands, demonstrating that the antenna can deliver strong and consistent performance over the intended spectrum. The antenna efficiency of the proposed design is presented in Figure 11b. The results demonstrate good radiation efficiency across the two operating frequency bands. Specifically, the antenna achieves an efficiency of approximately 87% at 28 GHz and 82% at 38 GHz, confirming that the antenna effectively converts the input power into radiated power with minimal losses. These results indicate that the implemented structural modifications and feeding technique maintain high performance while supporting the desired dual-band circular polarization operation.

**Figure 12:**
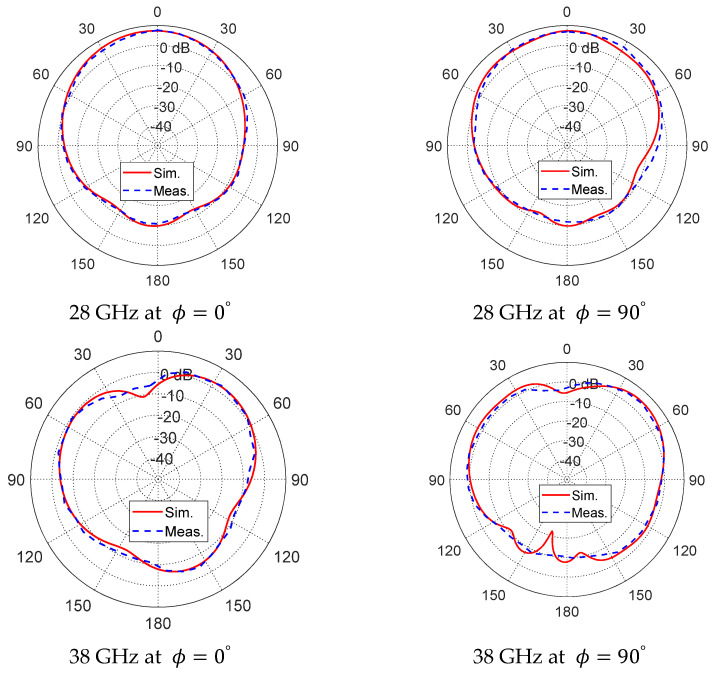
Simulated and measured radiation patterns of the antenna at both frequencies 28 and 38 GHz in the $\phi =0^{{}^{\circ}}$ and $\phi =90^{{}^{\circ}}$ planes.

In addition to gain, the directional characteristics of the antenna were carefully examined through far-field radiation pattern measurements at both resonant frequencies. Measurements were conducted in the principal planes, specifically at $\phi =0^{{}^{\circ}}$ and $\phi =90^{{}^{\circ}}$, and the results are depicted in Figure 12. The radiation patterns at 28 GHz and 38 GHz show nearly broadside directional radiation in both planes, with the measured results closely following the simulated patterns, indicating very good agreement between simulation and experiment. The patterns confirm that the antenna maintains a stable and predictable radiation behavior, with minimal distortion or asymmetry in the main lobe, ensuring reliable performance for circularly polarized applications. Overall, these results demonstrate that the proposed antenna not only achieves high gain at both target frequencies but also preserves consistent radiation characteristics across both planes, validating its effectiveness and suitability for dual-band millimeter-wave communications where precise directional control and circular polarization are critical. Moreover, the radiation efficiency of the proposed antenna is notably high at both operating bands, reaching approximately 89% at 28 GHz and 81% at 38 GHz, as shown in Figure 6b, which further confirms that the antenna effectively converts the accepted input power into radiated energy with minimal losses, reinforcing its suitability for efficient dual-band millimeter-wave operation.

**Figure 13:**
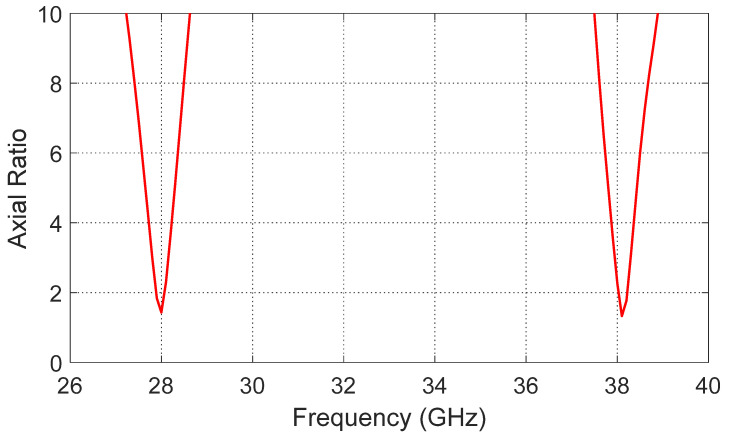
Axial ratio of the proposed modified quatrefoil antenna, demonstrating circular polarization with values below 3 dB at 28 GHz and 38 GHz.

Additionally, the antenna’s circular polarization performance is evaluated through the axial ratio, as shown in Figure 13, where values below 3 dB are observed at both resonant frequencies, specifically 1.4 dB at 28 GHz and 2.2 dB at 38 GHz, demonstrating high-quality circular polarization in both bands.

## 4. Surface Current Distribution for CP Behavior

The surface current distribution plays a crucial role in explaining and verifying the circular polarization behavior of the proposed antenna. To clearly demonstrate this mechanism, the surface currents are examined at four successive phase instants of the excitation cycle, namely at 0°, 90°, 180°, and 270°. At these four time steps, the direction and orientation of the surface current vectors rotate progressively along the modified quatrefoil-shaped radiator, forming a complete circular cycle. The arrows representing the surface current distribution indicate a continuous rotation of the current with time, which directly implies a rotating electric field in the far field and, consequently, the generation of circular polarization. This rotational behavior of the surface currents is observed at both operating frequencies of 28 GHz and 38 GHz, confirming that circular polarization is achieved in both bands. However, a clear difference can be identified in the current distribution patterns at the two frequencies. At 28 GHz, the surface current exhibits a first-order mode, characterized by a single dominant current loop with a smooth and uniform rotation. In contrast, at 38 GHz, although the surface current still shows a clear circular rotation confirming circular polarization, the distribution corresponds to a second-order mode, where additional current variations and nulls appear across the radiator. This higher-order behavior at 38 GHz explains the difference in modal operation between the two bands while still maintaining circular polarization, and it highlights the effectiveness of the modified quatrefoil geometry in supporting circularly polarized radiation through different resonant modes.

**Figure 14:**
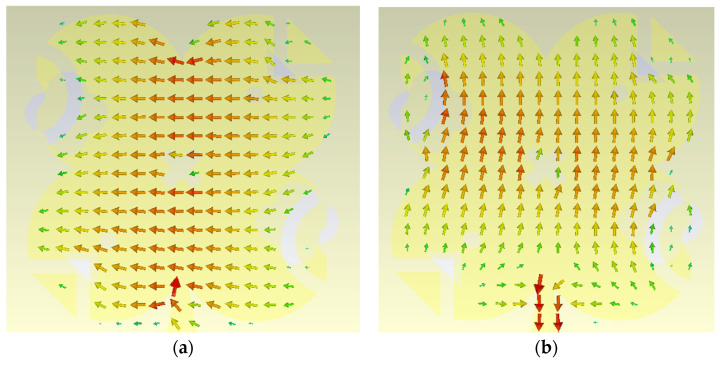

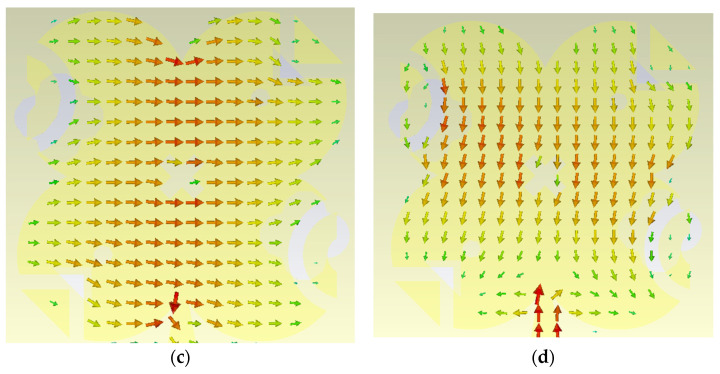
Surface current distribution of the proposed antenna at 28 GHz showing first-order mode behavior at four phase instants, (**a**) 0°, (**b**) 90°, (**c**) 180°, and (**d**) 270°, over one circular polarization cycle.

**Figure 15:**
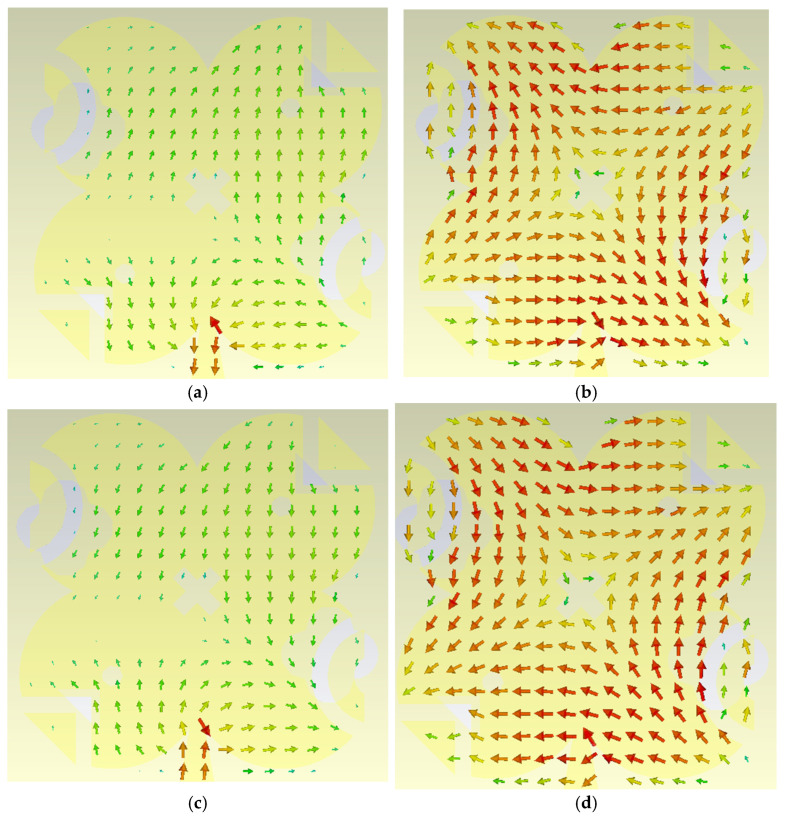
Surface current distribution of the proposed antenna at 38 GHz showing second-order mode behavior at four phase instants, (**a**) 0°, (**b**) 90°, (**c**) 180°, and (**d**) 270°, over one circular polarization cycle.

As illustrated in Figure 14, the surface current distribution at 28 GHz corresponds to a first-order mode, where four distinct current snapshots represent the four quarter-cycle phases (0°, 90°, 180°, and 270°) of the circular polarization cycle. The current rotates smoothly and uniformly around the radiator, indicating a dominant fundamental mode. Similarly, Figure 15 shows the surface current distributions at 38 GHz for the same four phase instants. Although the current rotation still clearly demonstrates circular polarization, the distribution at 38 GHz exhibits a second-order mode, characterized by more complex current paths and additional variations across the radiator. These observations confirm that the proposed modified quatrefoil antenna supports circular polarization at both operating frequencies, while operating in different resonant modes at 28 GHz and 38 GHz.

## 5. Comparison with Published Work

To highlight the effectiveness of the proposed antenna, its performance is systematically compared with several recent dual-band 28/38 GHz antenna designs reported in the literature, focusing on key metrics such as antenna size, operating bandwidth, axial ratio, radiation efficiency, and realized gain at each operating band, as summarized in Table 3.

Table 3 presents a comparison between the proposed antenna and previously reported circularly polarized millimeter-wave antennas. The proposed design demonstrates a highly compact size of 3.34 × 3.34 mm^2^, which is significantly smaller than the antennas reported in previous works. Despite this compact size, the antenna achieves realized gains of 7.5 dBi and 5.5 dBi at 28 GHz and 38 GHz, respectively, which are comparable to or higher than several reported designs. The antenna also maintains good radiation efficiencies of 87% and 82%, indicating effective radiation performance for millimeter-wave operation. In addition, the proposed antenna exhibits excellent circular polarization performance, with axial ratios of 1.4 dB and 2.2 dB, which are well below the 3-dB criterion and therefore provide stable CP radiation. The impedance bandwidths of 3.5% and 2.2% sufficiently cover the targeted 5G mm-wave bands. Another important advantage of the proposed design is its simple fabrication, as the antenna is implemented using a single-layer structure with a one-step fabrication process, unlike many previous designs that require complex geometries or multilayer structures. Therefore, the proposed antenna offers an attractive combination of ultra-compact size, good gain, high efficiency, and simple fabrication, making it a promising candidate for compact 5G millimeter-wave devices, phased arrays, and integrated wireless communication systems.

## 6. Conclusions

This work presented a compact dual-band circularly polarized antenna designed for millimeter-wave 5G applications operating at 28 GHz and 38 GHz. The proposed single-element antenna occupies a very small footprint of 8 × 16 mm^2^ and is implemented on a Rogers RO3003 substrate, making it well suited for space-constrained user equipment. Circular polarization is successfully achieved at both operating bands with low axial-ratio values, ensuring robustness against polarization mismatch and device orientation variations. The antenna demonstrates good impedance matching and stable radiation characteristics, with measured realized gains of 7.5 dBi at 28 GHz and 5.5 dBi at 38 GHz, along with high radiation efficiencies of 87% and 82%, respectively. Compared with recently reported designs in the literature, the proposed antenna offers a favourable trade-off between compact size, polarization performance, efficiency, and gain without relying on bulky reflectors or complex metasurface structures. These characteristics make the proposed design a promising candidate for future 5G and millimeter-wave wireless communication systems.

## Figures and Tables

**Table 1:** Optimized Dimensional Parameters of the Proposed Antenna.

**Parameter**	$W_{f}$	$L_{f1}$	$L_{f2}$	$L_{f3}$	$L_{f4}$	$d_{s}$	$d_{t1}$	$R_{L}$	$R_{Q}$
**Value (mm)**	0.64	1.4	3.3	0.6	6.6	0.2	0.71	0.6	0.88
**Parameter**	$S_{Q}$	$d_{t2}$	$L_{s}$	$W_{s}$	$L_{QG}$	$L_{t}$	$L_{ts}$	$S_{g1}$	$S_{g2}$
**Value (mm)**	2.26	0.66	16	8	0.92	3.92	2.08	2.04	1.8

**Table 2:** List of the devices used for experimental measurements of the antenna radiation characteristics.

Device	Model	Frequency Range
Vector Network Analyzer (VNA)	Rhode&Schwartz ZVA67	10 MHz–67 GHz
Vector Signal Generator (VSG)	Agilent E8267D	100 KHz–44 GHz
Vector Signal Analyzer (VSA)	Agilent N9010A	10 Hz–44 GHz
Reference Horn Antenna	A-Info LB018400	18–40 GHz
VNA Calibration kit	Rhode&Schwartz ZV-Z218	10 MHz–67 GHz

**Table 3:** Comparison of the proposed dual-band 28/38 GHz circularly polarized antenna with recent antenna designs reported in the literature.

	Antenna Size (mm × mm)	Gain at 28/38 (dBi)	Efficiency at 28/38 (%)	Axial Ratio	Impedance BW (%)	Fabrication Complexity
[9]	6.8 × 6.8	5.7/5.1	88/90	2.6/2.9	5.25/10.5	Low-Medium
[10]	26.4 × 20.4	7.37 × 7.06	94/96	2.7/2.9	2.5/3.2	Medium
[11]	11 × 14	4.3/6.26	93/89	1.5/2.5	14/5.01	Medium
[12]	3.1 × 3.5	4/4.5	87/91	1.5/1.7	3/1.9	Medium
Present Work	3.34 × 3.34	7.5/5.5	87/82	1.4/2.2	3.5/2.2	Low

## Data Availability

The data available to interested researchers upon request.
